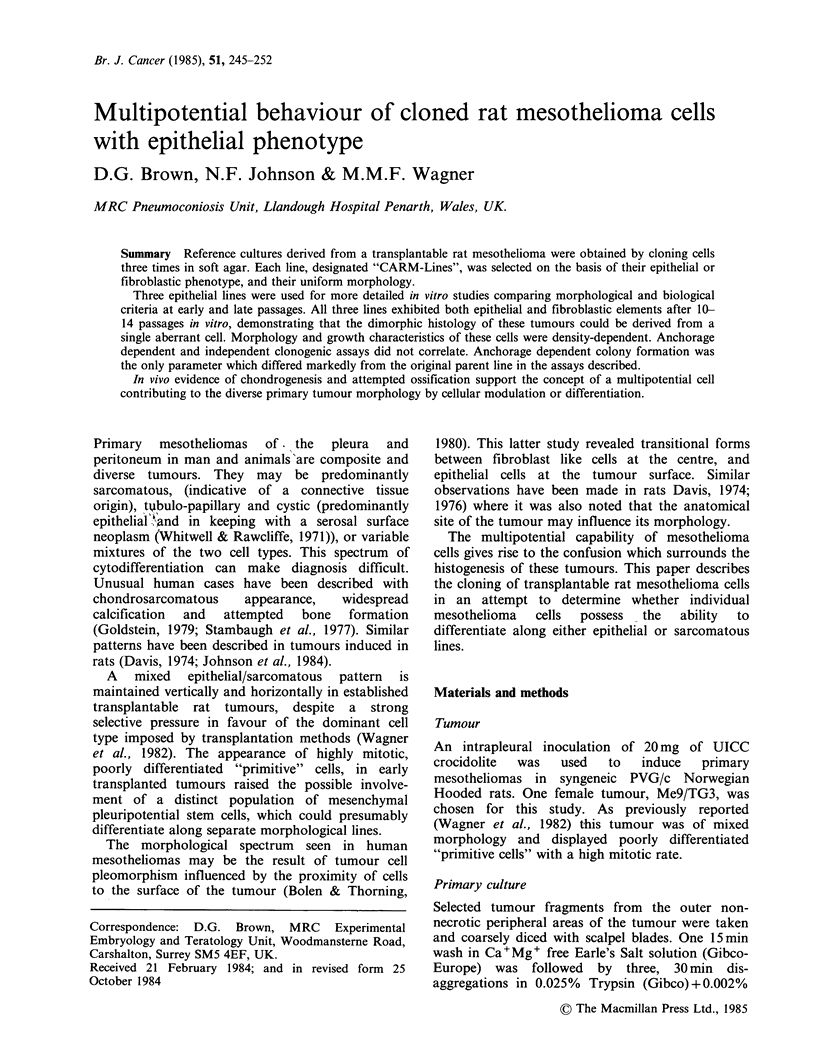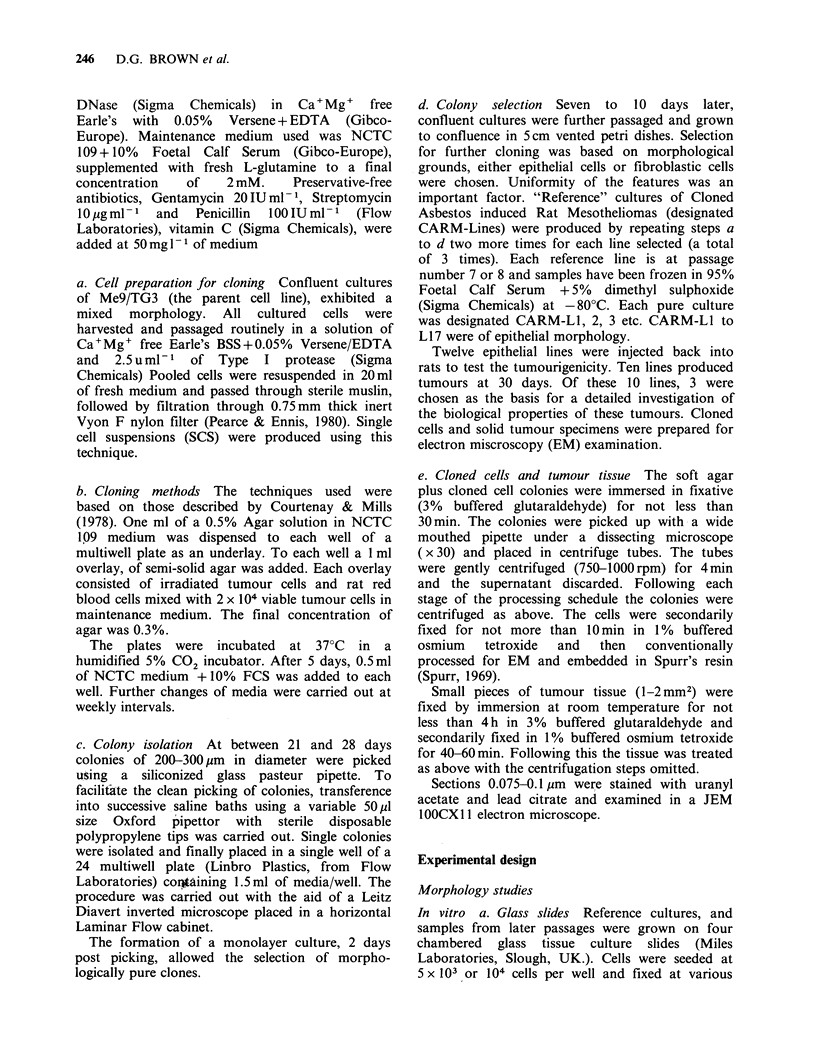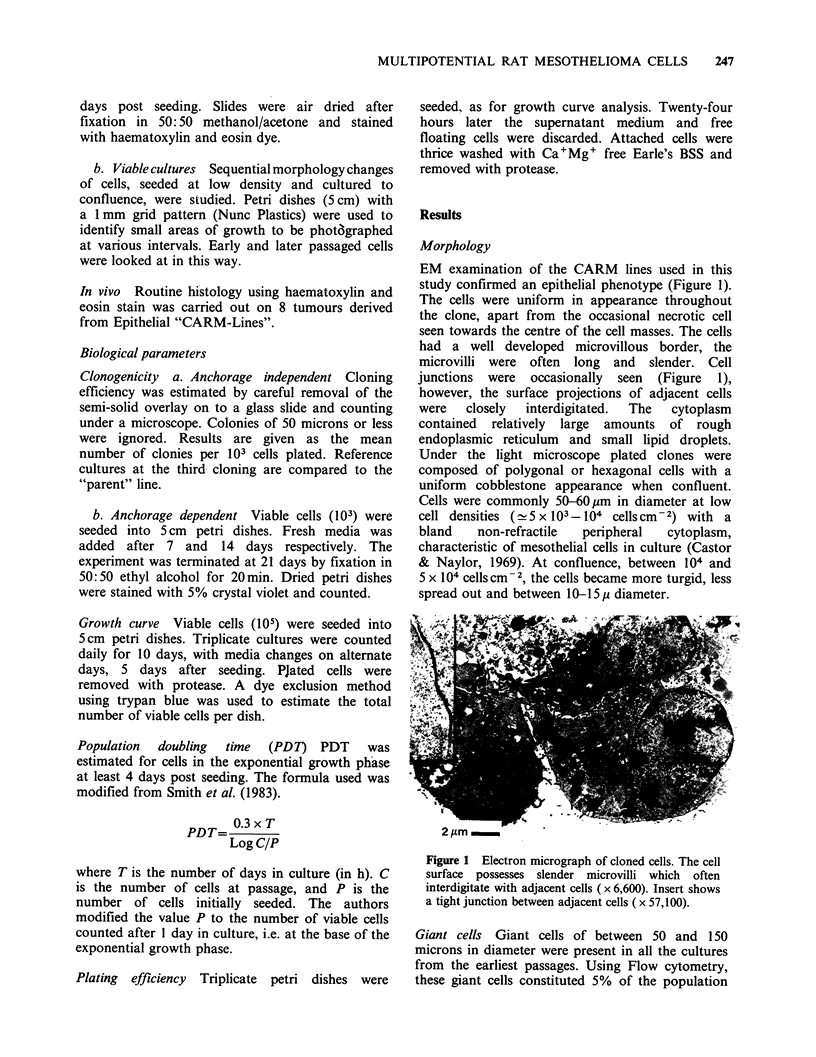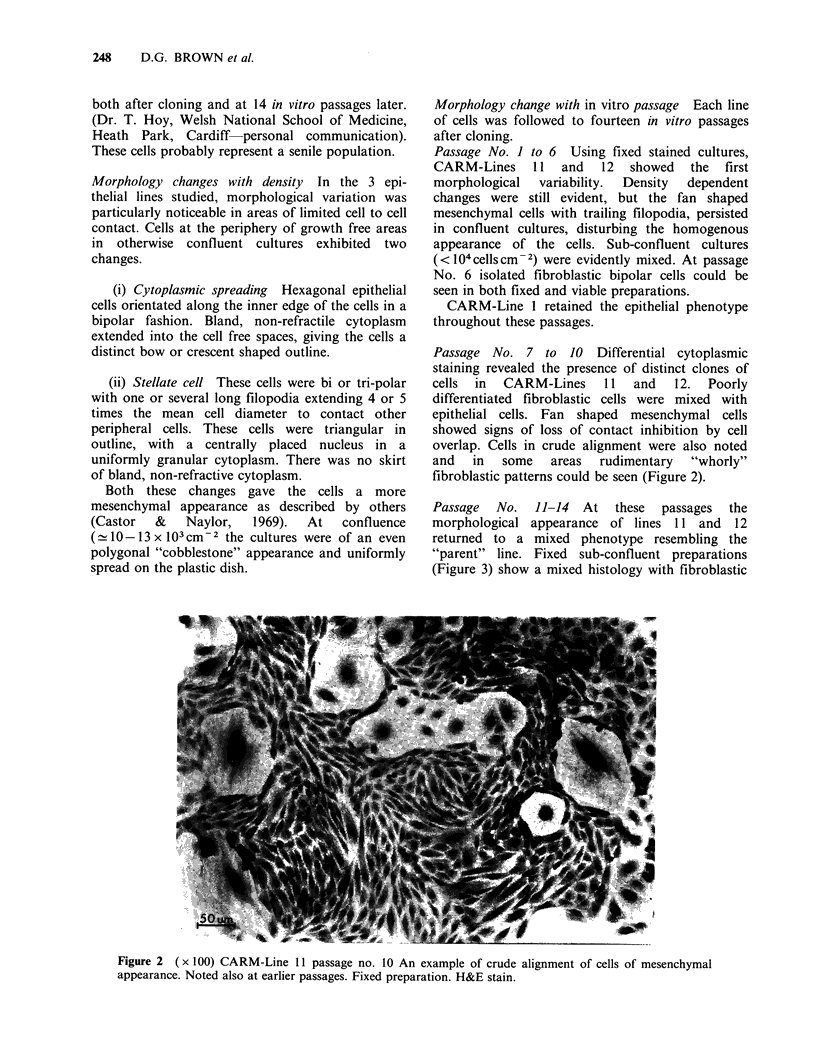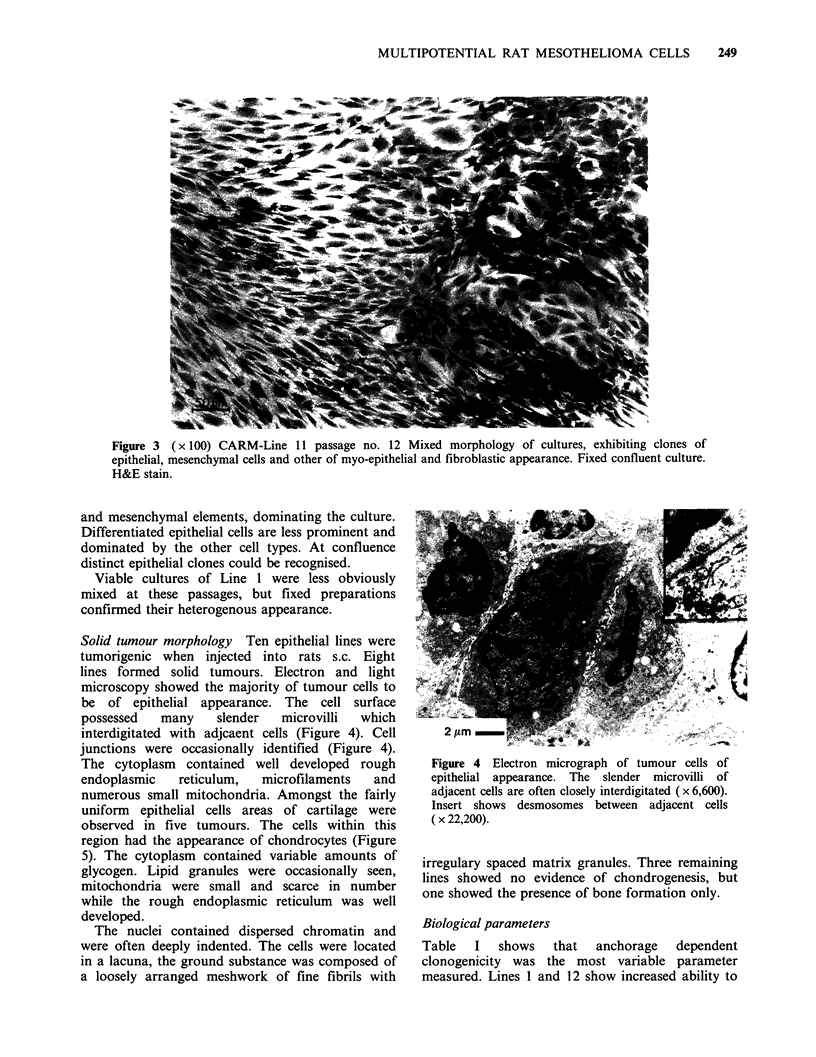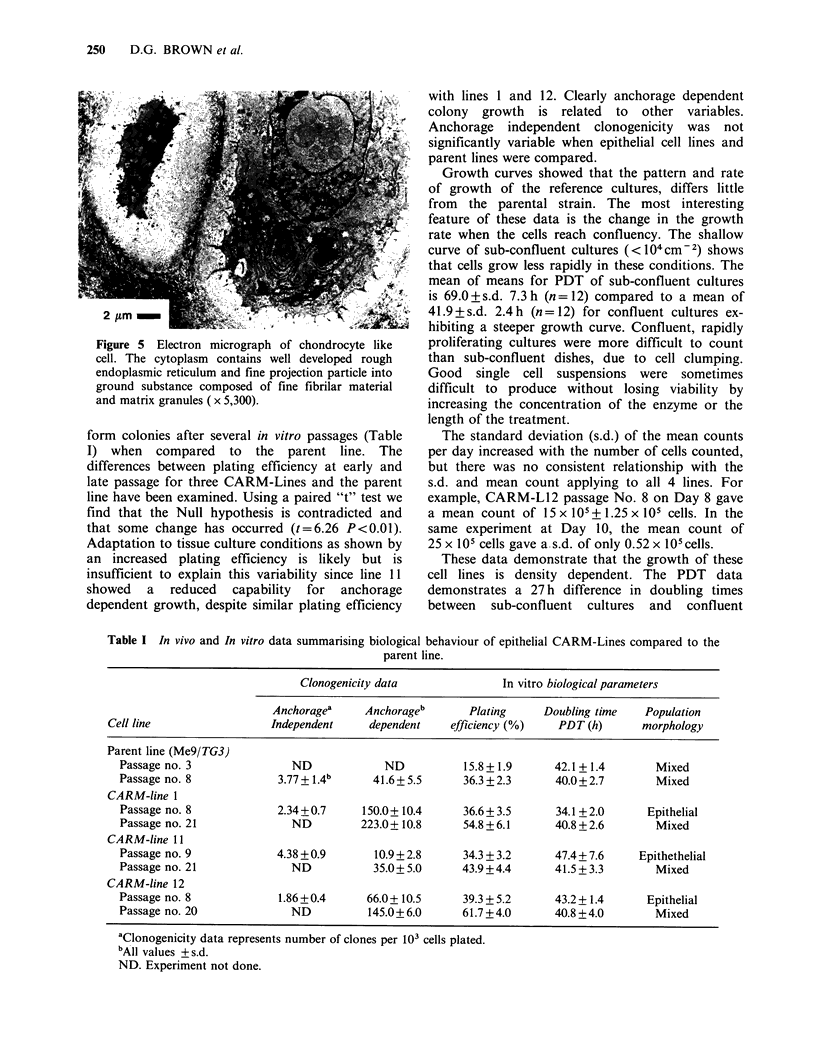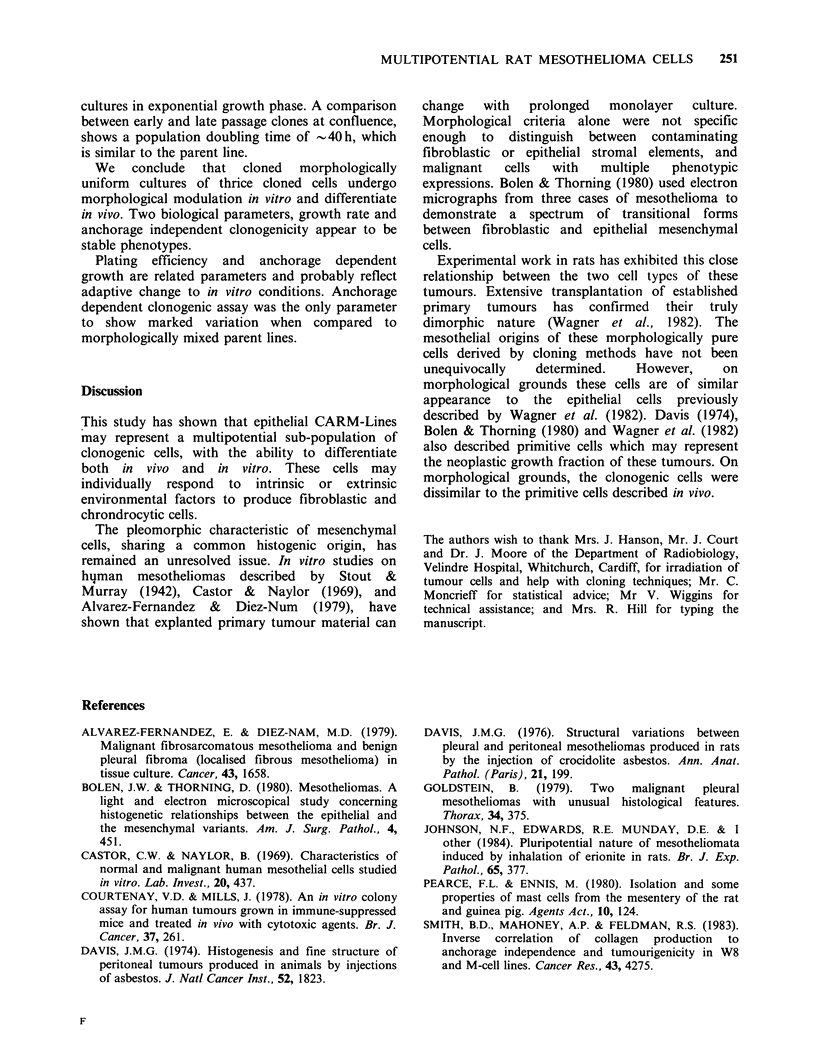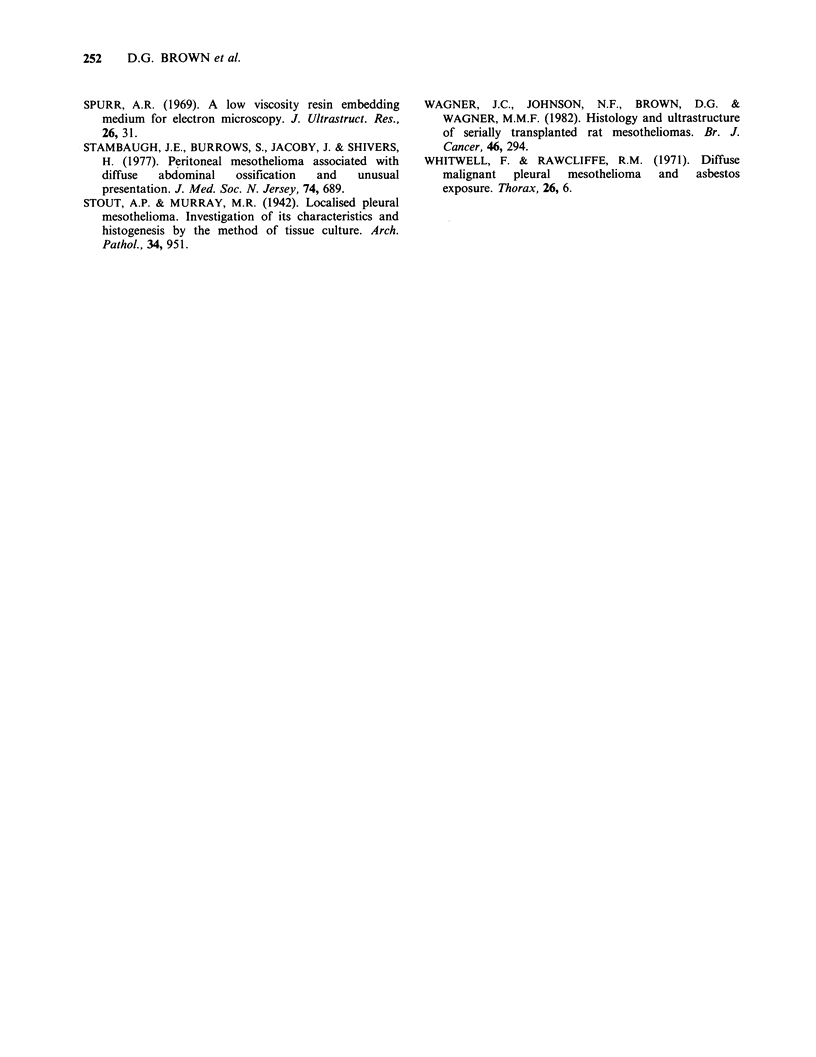# Multipotential behaviour of cloned rat mesothelioma cells with epithelial phenotype.

**DOI:** 10.1038/bjc.1985.35

**Published:** 1985-02

**Authors:** D. G. Brown, N. F. Johnson, M. M. Wagner

## Abstract

**Images:**


*Br. J. Cancer* (1985), **51**, 245–252

### Multipotential behaviour of cloned rat mesothelioma cells with epithelial phenotype

D.G. Brown, N.F. Johnson & M.M.F. Wagner

*MRC Pneumoconiosis Unit, Llandough Hospital Penarth, Wales, UK.*

**Summary** Reference cultures derived from a transplantable rat mesothelioma were obtained by cloning cells three times in soft agar. Each line, designated "CARM-Lines", was selected on the basis of their epithelial or fibroblastic phenotype, and their uniform morphology.

Three epithelial lines were used for more detailed *in vitro* studies comparing morphological and biological criteria at early and late passages. All three lines exhibited both epithelial and fibroblastic elements after 10–14 passages *in vitro*, demonstrating that the dimorphic histology of these tumours could be derived from a single aberrant cell. Morphology and growth characteristics of these cells were density-dependent. Anchorage dependent and independent clonogenic assays did not correlate. Anchorage dependent colony formation was the only parameter which differed markedly from the original parent line in the assays described.

*In vivo* evidence of chondrogenesis and attempted ossification support the concept of a multipotential cell contributing to the diverse primary tumour morphology by cellular modulation or differentiation.

Primary mesotheliomas of the pleura and peritoneum in man and animals are composite and diverse tumours. They may be predominantly sarcomatous, (indicative of a connective tissue origin), tubulo-papillary and cystic (predominantly epithelial and in keeping with a serosal surface neoplasm (Whitwell & Rawcliffe, 1971)), or variable mixtures of the two cell types. This spectrum of cytodifferentiation can make diagnosis difficult. Unusual human cases have been described with chondrosarcomatous appearance, widespread calcification and attempted bone formation (Goldstein, 1979; Stambaugh *et al.*, 1977). Similar patterns have been described in tumours induced in rats (Davis, 1974; Johnson *et al.*, 1984).

A mixed epithelial/sarcomatous pattern is maintained vertically and horizontally in established transplantable rat tumours, despite a strong selective pressure in favour of the dominant cell type imposed by transplantation methods (Wagner *et al.*, 1982). The appearance of highly mitotic, poorly differentiated "primitive" cells, in early transplanted tumours raised the possible involvement of a distinct population of mesenchymal pleuripotential stem cells, which could presumably differentiate along separate morphological lines.

The morphological spectrum seen in human mesotheliomas may be the result of tumour cell pleomorphism influenced by the proximity of cells to the surface of the tumour (Bolen & Thorning, 1980). This latter study revealed transitional forms between fibroblast like cells at the centre, and epithelial cells at the tumour surface. Similar observations have been made in rats Davis, 1974; 1976) where it was also noted that the anatomical site of the tumour may influence its morphology.

The multipotential capability of mesothelioma cells gives rise to the confusion which surrounds the histogenesis of these tumours. This paper describes the cloning of transplantable rat mesothelioma cells in an attempt to determine whether individual mesothelioma cells possess the ability to differentiate along either epithelial or sarcomatous lines.

### Materials and methods

### *Tumour*

An intrapleural inoculation of 20 mg of UICC crocidolite was used to induce primary mesotheliomas in syngeneic PVG/c Norwegian Hooded rats. One female tumour, Me9/TG3, was chosen for this study. As previously reported (Wagner *et al.*, 1982) this tumour was of mixed morphology and displayed poorly differentiated "primitive cells" with a high mitotic rate.

### *Primary culture*

Selected tumour fragments from the outer non-necrotic peripheral areas of the tumour were taken and coarsely diced with scalpel blades. One 15 min wash in $Ca^{+}Mg^{+}$ free Earle's Salt solution (Gibco-Europe) was followed by three, 30 min disaggregations in 0.025% Trypsin (Gibco) + 0.002%

Correspondence: D.G. Brown, MRC Experimental Embryology and Teratology Unit, Woodmansterne Road, Carshalton, Surrey SM5 4EF, UK.

Received 21 February 1984; and in revised form 25 October 1984

© The Macmillan Press Ltd., 1985

DNase (Sigma Chemicals) in $Ca^+Mg^+$ free Earle's with 0.05% Versene+EDTA (Gibco-Europe). Maintenance medium used was NCTC 109+10% Foetal Calf Serum (Gibco-Europe), supplemented with fresh L-glutamine to a final concentration of 2 mM. Preservative-free antibiotics, Gentamycin 20 IU $ml^{-1}$, Streptomycin 10 $\mu g\ ml^{-1}$ and Penicillin 100 IU $ml^{-1}$ (Flow Laboratories), vitamin C (Sigma Chemicals), were added at 50 mg $l^{-1}$ of medium

*a. Cell preparation for cloning* Confluent cultures of Me9/TG3 (the parent cell line), exhibited a mixed morphology. All cultured cells were harvested and passaged routinely in a solution of $Ca^+Mg^+$ free Earle's BSS+0.05% Versene/EDTA and 2.5 u $ml^{-1}$ of Type I protease (Sigma Chemicals) Pooled cells were resuspended in 20 ml of fresh medium and passed through sterile muslin, followed by filtration through 0.75 mm thick inert Vyon F nylon filter (Pearce & Ennis, 1980). Single cell suspensions (SCS) were produced using this technique.

*b. Cloning methods* The techniques used were based on those described by Courtenay & Mills (1978). One ml of a 0.5% Agar solution in NCTC 109 medium was dispensed to each well of a multiwell plate as an underlay. To each well a 1 ml overlay, of semi-solid agar was added. Each overlay consisted of irradiated tumour cells and rat red blood cells mixed with $2 \times 10^4$ viable tumour cells in maintenance medium. The final concentration of agar was 0.3%.

The plates were incubated at 37°C in a humidified 5% $CO_2$ incubator. After 5 days, 0.5 ml of NCTC medium +10% FCS was added to each well. Further changes of media were carried out at weekly intervals.

*c. Colony isolation* At between 21 and 28 days colonies of 200–300 $\mu m$ in diameter were picked using a siliconized glass pasteur pipette. To facilitate the clean picking of colonies, transference into successive saline baths using a variable 50 $\mu l$ size Oxford pipettor with sterile disposable polypropylene tips was carried out. Single colonies were isolated and finally placed in a single well of a 24 multiwell plate (Linbro Plastics, from Flow Laboratories) containing 1.5 ml of media/well. The procedure was carried out with the aid of a Leitz Diavert inverted microscope placed in a horizontal Laminar Flow cabinet.

The formation of a monolayer culture, 2 days post picking, allowed the selection of morphologically pure clones.

*d. Colony selection* Seven to 10 days later, confluent cultures were further passaged and grown to confluence in 5 cm vented petri dishes. Selection for further cloning was based on morphological grounds, either epithelial cells or fibroblastic cells were chosen. Uniformity of the features was an important factor. "Reference" cultures of Cloned Asbestos induced Rat Mesotheliomas (designated CARM-Lines) were produced by repeating steps *a* to *d* two more times for each line selected (a total of 3 times). Each reference line is at passage number 7 or 8 and samples have been frozen in 95% Foetal Calf Serum +5% dimethyl sulphoxide (Sigma Chemicals) at −80°C. Each pure culture was designated CARM-L1, 2, 3 etc. CARM-L1 to L17 were of epithelial morphology.

Twelve epithelial lines were injected back into rats to test the tumourigenicity. Ten lines produced tumours at 30 days. Of these 10 lines, 3 were chosen as the basis for a detailed investigation of the biological properties of these tumours. Cloned cells and solid tumour specimens were prepared for electron miscroscopy (EM) examination.

*e. Cloned cells and tumour tissue* The soft agar plus cloned cell colonies were immersed in fixative (3% buffered glutaraldehyde) for not less than 30 min. The colonies were picked up with a wide mouthed pipette under a dissecting microscope (×30) and placed in centrifuge tubes. The tubes were gently centrifuged (750–1000 rpm) for 4 min and the supernatant discarded. Following each stage of the processing schedule the colonies were centrifuged as above. The cells were secondarily fixed for not more than 10 min in 1% buffered osmium tetroxide and then conventionally processed for EM and embedded in Spurr's resin (Spurr, 1969).

Small pieces of tumour tissue (1–2 $mm^2$) were fixed by immersion at room temperature for not less than 4 h in 3% buffered glutaraldehyde and secondarily fixed in 1% buffered osmium tetroxide for 40–60 min. Following this the tissue was treated as above with the centrifugation steps omitted.

Sections 0.075–0.1 $\mu m$ were stained with uranyl acetate and lead citrate and examined in a JEM 100CX11 electron microscope.

### Experimental design

### *Morphology studies*

*In vitro a. Glass slides* Reference cultures, and samples from later passages were grown on four chambered glass tissue culture slides (Miles Laboratories, Slough, UK.). Cells were seeded at $5 \times 10^3$ or $10^4$ cells per well and fixed at various days post seeding. Slides were air dried after fixation in 50:50 methanol/acetone and stained with haematoxylin and eosin dye.

*b. Viable cultures* Sequential morphology changes of cells, seeded at low density and cultured to confluence, were studied. Petri dishes (5 cm) with a 1 mm grid pattern (Nunc Plastics) were used to identify small areas of growth to be photographed at various intervals. Early and later passaged cells were looked at in this way.

*In vivo* Routine histology using haematoxylin and eosin stain was carried out on 8 tumours derived from Epithelial "CARM-Lines".

*Biological parameters*

*Clonogenicity a. Anchorage independent* Cloning efficiency was estimated by careful removal of the semi-solid overlay on to a glass slide and counting under a microscope. Colonies of 50 microns or less were ignored. Results are given as the mean number of clonies per $10^3$ cells plated. Reference cultures at the third cloning are compared to the "parent" line.

*b. Anchorage dependent* Viable cells ($10^3$) were seeded into 5 cm petri dishes. Fresh media was added after 7 and 14 days respectively. The experiment was terminated at 21 days by fixation in 50:50 ethyl alcohol for 20 min. Dried petri dishes were stained with 5% crystal violet and counted.

*Growth curve* Viable cells ($10^5$) were seeded into 5 cm petri dishes. Triplicate cultures were counted daily for 10 days, with media changes on alternate days, 5 days after seeding. Plated cells were removed with protease. A dye exclusion method using trypan blue was used to estimate the total number of viable cells per dish.

*Population doubling time (PDT)* PDT was estimated for cells in the exponential growth phase at least 4 days post seeding. The formula used was modified from Smith *et al.* (1983).

$$PDT = \frac{0.3 \times T}{\text{Log}\, C/P}$$

where $T$ is the number of days in culture (in h). $C$ is the number of cells at passage, and $P$ is the number of cells initially seeded. The authors modified the value $P$ to the number of viable cells counted after 1 day in culture, i.e. at the base of the exponential growth phase.

*Plating efficiency* Triplicate petri dishes were seeded, as for growth curve analysis. Twenty-four hours later the supernatant medium and free floating cells were discarded. Attached cells were thrice washed with $Ca^+Mg^+$ free Earle's BSS and removed with protease.

### Results

*Morphology*

EM examination of the CARM lines used in this study confirmed an epithelial phenotype (Figure 1). The cells were uniform in appearance throughout the clone, apart from the occasional necrotic cell seen towards the centre of the cell masses. The cells had a well developed microvillous border, the microvilli were often long and slender. Cell junctions were occasionally seen (Figure 1), however, the surface projections of adjacent cells were closely interdigitated. The cytoplasm contained relatively large amounts of rough endoplasmic reticulum and small lipid droplets. Under the light microscope plated clones were composed of polygonal or hexagonal cells with a uniform cobblestone appearance when confluent. Cells were commonly 50–60 $\mu$m in diameter at low cell densities ($\simeq 5 \times 10^3 - 10^4$ cells cm$^{-2}$) with a bland non-refractile peripheral cytoplasm, characteristic of mesothelial cells in culture (Castor & Naylor, 1969). At confluence, between $10^4$ and $5 \times 10^4$ cells cm$^{-2}$, the cells became more turgid, less spread out and between 10–15 $\mu$ diameter.

**Figure 1** Electron micrograph of cloned cells. The cell surface possesses slender microvilli which often interdigitate with adjacent cells ( $\times$ 6,600). Insert shows a tight junction between adjacent cells ( $\times$ 57,100).

*Giant cells* Giant cells of between 50 and 150 microns in diameter were present in all the cultures from the earliest passages. Using Flow cytometry, these giant cells constituted 5% of the population both after cloning and at 14 *in vitro* passages later. (Dr. T. Hoy, Welsh National School of Medicine, Heath Park, Cardiff—personal communication). These cells probably represent a senile population.

*Morphology changes with density* In the 3 epithelial lines studied, morphological variation was particularly noticeable in areas of limited cell to cell contact. Cells at the periphery of growth free areas in otherwise confluent cultures exhibited two changes.

(i) *Cytoplasmic spreading* Hexagonal epithelial cells orientated along the inner edge of the cells in a bipolar fashion. Bland, non-refractile cytoplasm extended into the cell free spaces, giving the cells a distinct bow or crescent shaped outline.

(ii) *Stellate cell* These cells were bi or tri-polar with one or several long filopodia extending 4 or 5 times the mean cell diameter to contact other peripheral cells. These cells were triangular in outline, with a centrally placed nucleus in a uniformly granular cytoplasm. There was no skirt of bland, non-refractive cytoplasm.

Both these changes gave the cells a more mesenchymal appearance as described by others (Castor & Naylor, 1969). At confluence ($\simeq 10-13 \times 10^3 \text{cm}^{-2}$ the cultures were of an even polygonal "cobblestone" appearance and uniformly spread on the plastic dish.

*Morphology change with* in vitro *passage* Each line of cells was followed to fourteen *in vitro* passages after cloning.

*Passage No. 1 to 6* Using fixed stained cultures, CARM-Lines 11 and 12 showed the first morphological variability. Density dependent changes were still evident, but the fan shaped mesenchymal cells with trailing filopodia, persisted in confluent cultures, disturbing the homogenous appearance of the cells. Sub-confluent cultures ($< 10^4 \text{cells cm}^{-2}$) were evidently mixed. At passage No. 6 isolated fibroblastic bipolar cells could be seen in both fixed and viable preparations.

CARM-Line 1 retained the epithelial phenotype throughout these passages.

*Passage No. 7 to 10* Differential cytoplasmic staining revealed the presence of distinct clones of cells in CARM-Lines 11 and 12. Poorly differentiated fibroblastic cells were mixed with epithelial cells. Fan shaped mesenchymal cells showed signs of loss of contact inhibition by cell overlap. Cells in crude alignment were also noted and in some areas rudimentary "whorly" fibroblastic patterns could be seen (Figure 2).

*Passage No. 11–14* At these passages the morphological appearance of lines 11 and 12 returned to a mixed phenotype resembling the "parent" line. Fixed sub-confluent preparations (Figure 3) show a mixed histology with fibroblastic

**Figure 2** (×100) CARM-Line 11 passage no. 10 An example of crude alignment of cells of mesenchymal appearance. Noted also at earlier passages. Fixed preparation. H&E stain.

**Figure 3** (×100) CARM-Line 11 passage no. 12 Mixed morphology of cultures, exhibiting clones of epithelial, mesenchymal cells and other of myo-epithelial and fibroblastic appearance. Fixed confluent culture. H&E stain.

and mesenchymal elements, dominating the culture. Differentiated epithelial cells are less prominent and dominated by the other cell types. At confluence distinct epithelial clones could be recognised.

Viable cultures of Line 1 were less obviously mixed at these passages, but fixed preparations confirmed their heterogenous appearance.

*Solid tumour morphology* Ten epithelial lines were tumorigenic when injected into rats s.c. Eight lines formed solid tumours. Electron and light microscopy showed the majority of tumour cells to be of epithelial appearance. The cell surface possessed many slender microvilli which interdigitated with adjcaent cells (Figure 4). Cell junctions were occasionally identified (Figure 4). The cytoplasm contained well developed rough endoplasmic reticulum, microfilaments and numerous small mitochondria. Amongst the fairly uniform epithelial cells areas of cartilage were observed in five tumours. The cells within this region had the appearance of chondrocytes (Figure 5). The cytoplasm contained variable amounts of glycogen. Lipid granules were occasionally seen, mitochondria were small and scarce in number while the rough endoplasmic reticulum was well developed.

The nuclei contained dispersed chromatin and were often deeply indented. The cells were located in a lacuna, the ground substance was composed of a loosely arranged meshwork of fine fibrils with

**Figure 4** Electron micrograph of tumour cells of epithelial appearance. The slender microvilli of adjacent cells are often closely interdigitated (×6,600). Insert shows desmosomes between adjacent cells (×22,200).

irregulary spaced matrix granules. Three remaining lines showed no evidence of chondrogenesis, but one showed the presence of bone formation only.

*Biological parameters*

Table I shows that anchorage dependent clonogenicity was the most variable parameter measured. Lines 1 and 12 show increased ability to

**Figure 5** Electron micrograph of chondrocyte like cell. The cytoplasm contains well developed rough endoplasmic reticulum and fine projection particle into ground substance composed of fine fibrilar material and matrix granules ( × 5,300).

form colonies after several *in vitro* passages (Table I) when compared to the parent line. The differences between plating efficiency at early and late passage for three CARM-Lines and the parent line have been examined. Using a paired "t" test we find that the Null hypothesis is contradicted and that some change has occurred ($t = 6.26$ $P < 0.01$). Adaptation to tissue culture conditions as shown by an increased plating efficiency is likely but is insufficient to explain this variability since line 11 showed a reduced capability for anchorage dependent growth, despite similar plating efficiency with lines 1 and 12. Clearly anchorage dependent colony growth is related to other variables. Anchorage independent clonogenicity was not significantly variable when epithelial cell lines and parent lines were compared.

Growth curves showed that the pattern and rate of growth of the reference cultures, differs little from the parental strain. The most interesting feature of these data is the change in the growth rate when the cells reach confluency. The shallow curve of sub-confluent cultures ($< 10^4 cm^{-2}$) shows that cells grow less rapidly in these conditions. The mean of means for PDT of sub-confluent cultures is $69.0 \pm$ s.d. 7.3 h ($n = 12$) compared to a mean of $41.9 \pm$ s.d. 2.4 h ($n = 12$) for confluent cultures exhibiting a steeper growth curve. Confluent, rapidly proliferating cultures were more difficult to count than sub-confluent dishes, due to cell clumping. Good single cell suspensions were sometimes difficult to produce without losing viability by increasing the concentration of the enzyme or the length of the treatment.

The standard deviation (s.d.) of the mean counts per day increased with the number of cells counted, but there was no consistent relationship with the s.d. and mean count applying to all 4 lines. For example, CARM-L12 passage No. 8 on Day 8 gave a mean count of $15 \times 10^5 \pm 1.25 \times 10^5$ cells. In the same experiment at Day 10, the mean count of $25 \times 10^5$ cells gave a s.d. of only $0.52 \times 10^5$ cells.

These data demonstrate that the growth of these cell lines is density dependent. The PDT data demonstrates a 27 h difference in doubling times between sub-confluent cultures and confluent

**Table I** *In vivo* and *In vitro* data summarising biological behaviour of epithelial CARM-Lines compared to the parent line.

| | *Clonogenicity data* | | In vitro *biological parameters* | | |
|---|---|---|---|---|---|
| *Cell line* | *Anchorage*[a] *Independent* | *Anchorage*[b] *dependent* | *Plating efficiency* (%) | *Doubling time PDT* (*h*) | *Population morphology* |
| Parent line (Me9/*TG3*) | | | | | |
| Passage no. 3 | ND | ND | $15.8 \pm 1.9$ | $42.1 \pm 1.4$ | Mixed |
| Passage no. 8 | $3.77 \pm 1.4$[b] | $41.6 \pm 5.5$ | $36.3 \pm 2.3$ | $40.0 \pm 2.7$ | Mixed |
| *CARM-line* 1 | | | | | |
| Passage no. 8 | $2.34 \pm 0.7$ | $150.0 \pm 10.4$ | $36.6 \pm 3.5$ | $34.1 \pm 2.0$ | Epithelial |
| Passage no. 21 | ND | $223.0 \pm 10.8$ | $54.8 \pm 6.1$ | $40.8 \pm 2.6$ | Mixed |
| *CARM-line* 11 | | | | | |
| Passage no. 9 | $4.38 \pm 0.9$ | $10.9 \pm 2.8$ | $34.3 \pm 3.2$ | $47.4 \pm 7.6$ | Epithethelial |
| Passage no. 21 | ND | $35.0 \pm 5.0$ | $43.9 \pm 4.4$ | $41.5 \pm 3.3$ | Mixed |
| *CARM-line* 12 | | | | | |
| Passage no. 8 | $1.86 \pm 0.4$ | $66.0 \pm 10.5$ | $39.3 \pm 5.2$ | $43.2 \pm 1.4$ | Epithelial |
| Passage no. 20 | ND | $145.0 \pm 6.0$ | $61.7 \pm 4.0$ | $40.8 \pm 4.0$ | Mixed |

[a]Clonogenicity data represents number of clones per $10^3$ cells plated.
[b]All values ±s.d.
ND. Experiment not done.

cultures in exponential growth phase. A comparison between early and late passage clones at confluence, shows a population doubling time of $\sim$40 h, which is similar to the parent line.

We conclude that cloned morphologically uniform cultures of thrice cloned cells undergo morphological modulation *in vitro* and differentiate *in vivo*. Two biological parameters, growth rate and anchorage independent clonogenicity appear to be stable phenotypes.

Plating efficiency and anchorage dependent growth are related parameters and probably reflect adaptive change to *in vitro* conditions. Anchorage dependent clonogenic assay was the only parameter to show marked variation when compared to morphologically mixed parent lines.

### Discussion

This study has shown that epithelial CARM-Lines may represent a multipotential sub-population of clonogenic cells, with the ability to differentiate both *in vivo* and *in vitro*. These cells may individually respond to intrinsic or extrinsic environmental factors to produce fibroblastic and chrondrocytic cells.

The pleomorphic characteristic of mesenchymal cells, sharing a common histogenic origin, has remained an unresolved issue. *In vitro* studies on human mesotheliomas described by Stout & Murray (1942), Castor & Naylor (1969), and Alvarez-Fernandez & Diez-Num (1979), have shown that explanted primary tumour material can change with prolonged monolayer culture. Morphological criteria alone were not specific enough to distinguish between contaminating fibroblastic or epithelial stromal elements, and malignant cells with multiple phenotypic expressions. Bolen & Thorning (1980) used electron micrographs from three cases of mesothelioma to demonstrate a spectrum of transitional forms between fibroblastic and epithelial mesenchymal cells.

Experimental work in rats has exhibited this close relationship between the two cell types of these tumours. Extensive transplantation of established primary tumours has confirmed their truly dimorphic nature (Wagner *et al.*, 1982). The mesothelial origins of these morphologically pure cells derived by cloning methods have not been unequivocally determined. However, on morphological grounds these cells are of similar appearance to the epithelial cells previously described by Wagner *et al.* (1982). Davis (1974), Bolen & Thorning (1980) and Wagner *et al.* (1982) also described primitive cells which may represent the neoplastic growth fraction of these tumours. On morphological grounds, the clonogenic cells were dissimilar to the primitive cells described *in vivo*.

The authors wish to thank Mrs. J. Hanson, Mr. J. Court and Dr. J. Moore of the Department of Radiobiology, Velindre Hospital, Whitchurch, Cardiff, for irradiation of tumour cells and help with cloning techniques; Mr. C. Moncrieff for statistical advice; Mr V. Wiggins for technical assistance; and Mrs. R. Hill for typing the manuscript.

### References

ALVAREZ-FERNANDEZ, E. & DIEZ-NAM, M.D. (1979). Malignant fibrosarcomatous mesothelioma and benign pleural fibroma (localised fibrous mesothelioma) in tissue culture. *Cancer*, **43**, 1658.

BOLEN, J.W. & THORNING, D. (1980). Mesotheliomas. A light and electron microscopical study concerning histogenetic relationships between the epithelial and the mesenchymal variants. *Am. J. Surg. Pathol.*, **4**, 451.

CASTOR, C.W. & NAYLOR, B. (1969). Characteristics of normal and malignant human mesothelial cells studied *in vitro*. *Lab. Invest.*, **20**, 437.

COURTENAY, V.D. & MILLS, J. (1978). An *in vitro* colony assay for human tumours grown in immune-suppressed mice and treated *in vivo* with cytotoxic agents. *Br. J. Cancer*, **37**, 261.

DAVIS, J.M.G. (1974). Histogenesis and fine structure of peritoneal tumours produced in animals by injections of asbestos. *J. Natl Cancer Inst.*, **52**, 1823.

DAVIS, J.M.G. (1976). Structural variations between pleural and peritoneal mesotheliomas produced in rats by the injection of crocidolite asbestos. *Ann. Anat. Pathol. (Paris)*, **21**, 199.

GOLDSTEIN, B. (1979). Two malignant pleural mesotheliomas with unusual histological features. *Thorax*, **34**, 375.

JOHNSON, N.F., EDWARDS, R.E. MUNDAY, D.E. & 1 other (1984). Pluripotential nature of mesotheliomata induced by inhalation of erionite in rats. *Br. J. Exp. Pathol.*, **65**, 377.

PEARCE, F.L. & ENNIS, M. (1980). Isolation and some properties of mast cells from the mesentery of the rat and guinea pig. *Agents Act.*, **10**, 124.

SMITH, B.D., MAHONEY, A.P. & FELDMAN, R.S. (1983). Inverse correlation of collagen production to anchorage independence and tumourigenicity in W8 and M-cell lines. *Cancer Res.*, **43**, 4275.

SPURR, A.R. (1969). A low viscosity resin embedding medium for electron microscopy. *J. Ultrastruct. Res.*, **26**, 31.

STAMBAUGH, J.E., BURROWS, S., JACOBY, J. & SHIVERS, H. (1977). Peritoneal mesothelioma associated with diffuse abdominal ossification and unusual presentation. *J. Med. Soc. N. Jersey*, **74**, 689.

STOUT, A.P. & MURRAY, M.R. (1942). Localised pleural mesothelioma. Investigation of its characteristics and histogenesis by the method of tissue culture. *Arch. Pathol.*, **34**, 951.

WAGNER, J.C., JOHNSON, N.F., BROWN, D.G. & WAGNER, M.M.F. (1982). Histology and ultrastructure of serially transplanted rat mesotheliomas. *Br. J. Cancer*, **46**, 294.

WHITWELL, F. & RAWCLIFFE, R.M. (1971). Diffuse malignant pleural mesothelioma and asbestos exposure. *Thorax*, **26**, 6.